# Measuring Vulnerability for Small Areas

**DOI:** 10.23889/ijpds.v9i5.2917

**Published:** 2024-09-18

**Authors:** Chase Sawyer

**Affiliations:** 1 Census Bureau

This presentation will show how the Census Bureau used multiple data sources and small area methodologies to create the Community Resilience Estimates, a measure of social vulnerability to disasters.

